# Potential Impact of the Resistance to Quaternary Ammonium Disinfectants on the Persistence of *Listeria monocytogenes* in Food Processing Environments

**DOI:** 10.3389/fmicb.2016.00638

**Published:** 2016-05-02

**Authors:** Joaquín V. Martínez-Suárez, Sagrario Ortiz, Victoria López-Alonso

**Affiliations:** ^1^Departamento de Tecnología de Alimentos, Instituto Nacional de Investigación y Tecnología Agraria y AlimentariaMadrid, Spain; ^2^Unidad de Biología Computacional, Unidad Funcional de Investigación de Enfermedades Crónicas, Instituto de Salud Carlos IIIMadrid, Spain

**Keywords:** *Listeria monocytogenes*, food processing, bacterial persistence, quaternary ammonium disinfectants, resistance

## Abstract

The persistence of certain strains of *Listeria monocytogenes*, even after the food processing environment has been cleaned and disinfected, suggests that this may be related to phenomena that reduce the concentration of the disinfectants to subinhibitory levels. This includes (i) the existence of environmental niches or reservoirs that are difficult for disinfectants to reach, (ii) microorganisms that form biofilms and create microenvironments in which adequate concentrations of disinfectants cannot be attained, and (iii) the acquisition of resistance mechanisms in *L. monocytogenes*, including those that lead to a reduction in the intracellular concentration of the disinfectants. The only available data with regard to the resistance of *L. monocytogenes* to disinfectants applied in food production environments refer to genotypic resistance to quaternary ammonium compounds (QACs). Although there are several well-characterized eﬄux pumps that confer resistance to QACs, it is a low-level resistance that does not generate resistance to QACs at the concentrations applied in the food industry. However, dilution in the environment and biodegradation result in QAC concentration gradients. As a result, the microorganisms are frequently exposed to subinhibitory concentrations of QACs. Therefore, the low-level resistance to QACs in *L. monocytogenes* may contribute to its environmental adaptation and persistence. In fact, in certain cases, the relationship between low-level resistance and the environmental persistence of *L. monocytogenes* in different food production chains has been previously established. The resistant strains would have survival advantages in these environments over sensitive strains, such as the ability to form biofilms in the presence of increased biocide concentrations.

## Introduction

*Listeria monocytogenes* is a Gram-positive foodborne pathogen that can cause listeriosis, a relatively uncommon illness with a 20–30% case fatality rate (Silk et al., 2012; CDC, 2014; de Noordhout et al., 2014; EFSA and ECDC, 2014). The majority of cases of human listeriosis are caused by contaminated processed foods (McLauchlin et al., 2004; Lianou and Sofos, 2007). Although the original source of the contamination may be the raw food materials used in the processing plants, the strains of *L. monocytogenes* present in the final food products are usually different from the strains in the raw materials (Tompkin, 2002; Thévenot et al., 2006). In addition, when listeriosis outbreaks have been investigated, contamination is usually traced to the processing environment and equipment (Orsi et al., 2008; Nakari et al., 2014). This suggests that the contamination mainly occurs during the processing of food and is primarily due to strains from the processing plant environment (Malley et al., 2015; Garner and Kathariou, 2016).

## Environmental Persistence

The molecular characterization of *L. monocytogenes* isolates from the food processing environment regularly shows the presence of a reduced number of molecular subtypes and the long-term persistence of specific strains that can contaminate food and cause foodborne listeriosis (Ortiz et al., 2010; Ferreira et al., 2011; Morganti et al., 2015). The detection of highly similar isolates from different areas inside single establishments, and their environmental persistence, is a matter of concern for the hygienic management of food establishments (Cramer, 2006; Ferreira et al., 2014).

Carpentier and Cerf (2011) assert that *L. monocytogenes* persistence is primarily a random process given that “there are no strains with unique properties that lead to persistence but harborage sites in food industry premises and equipment where *L. monocytogenes* can persist.” Persistent strains of *L. monocytogenes* have occasionally been isolated in food processing environments after cleaning and disinfection (Lundén et al., 2003b; Soumet et al., 2005; Thévenot et al., 2006; Bērziņš et al., 2010; Ortiz et al., 2016). As demonstrated by the findings of studies reviewed here, the persistence of certain strains of *L. monocytogenes* after cleaning and disinfecting is not a completely random process, but rather an event related to different situations that result in subinhibitory concentrations of disinfectants at different scales. This includes: (i) local environmental conditions that can lead to the formation of niches or reservoirs that are difficult for disinfectants to reach; (ii) microorganisms that form biofilms and create microenvironments in which adequate concentrations of disinfectants cannot be attained; and (iii) the acquisition of resistance mechanisms in *L. monocytogenes*, including those that lead to a reduction in the intracellular concentration of the disinfectants.

There are numerous factors in food processing plants, such as insufficient cleaning before disinfection, disinfection of wet surfaces, and dosage failure that can lead to subinhibitory concentrations of disinfectants, thus reducing their efficiency. This reduction may be especially significant in certain niches or reservoirs in which water and organic matter are abundant, creating an environment where bacteria can survive and multiply readily (Cramer, 2006; Carpentier and Cerf, 2011).

Failure to effectively clean and disinfect the processing plant environment may contribute to biofilm formation in certain niches, leading to bacterial persistence (Gandhi and Chikindas, 2007; Renier et al., 2011). Cells embedded in the biofilm matrix display an increased resistance to biocide treatments (Bridier et al., 2011). However, the resistance to disinfectants in sessile cells (biofilms) and in planktonic cells (free-floating) are clearly different phenomena (Fatemi and Frank, 1999; Norwood and Gilmour, 2000; Stopforth et al., 2002; Kastbjerg and Gram, 2009). Resistance of biofilms to disinfectants is considered to be a form of “phenotypic resistance” since bacterial resistance is mainly induced by a physiological adaptation to the biofilm mode of life and can be lost or markedly reduced when biofilm cells revert to the planktonic state (Pan et al., 2006; Bridier et al., 2011). In contrast, in the case of planktonic cells, a bacterial strain is defined as being resistant to a biocide if it is not inhibited by a specific concentration that usually inhibit the majority of other strains (Bridier et al., 2011); that is, planktonic cell resistance depends on intrinsic cellular attributes such as intra-species (i.e., strain) variability in the MIC of a given disinfectant (Kastbjerg and Gram, 2012).

## Resistance To Disinfectants

The only available data with regard to the resistance of *L. monocytogenes* to disinfectants applied in food production environments refer to genotypic resistance to QACs (Hegstad et al., 2010; Gerba, 2015). BAC is typically used in studies assessing *in vitro* the activity of QACs. Increased MICs of BAC have been found in *L. monocytogenes* strains from different food production chains in different countries (**Table 1**). BAC resistance in *L. monocytogenes* is a low-level resistance. This means that the resistant strains only have a two to eight-fold increase in the MIC compared to the rest of the strains (**Table 1**). This resistance does not lead to QAC resistance at the concentrations that are normally used in the food industry (typically 200–1000 mg L^-1^) (SCENIHR, 2009; Ferreira et al., 2014; Tezel and Pavlostathis, 2015). Therefore, QACs are considered to be an effective means of eliminating the resistant *L. monocytogenes* strains (Kastbjerg and Gram, 2009, 2012).

**Table 1:** Reports of resistance to BAC in planktonic cells of *Listeria monocytogenes.*

No. assayed isolates	No. resistant isolates (%)	MIC (mg L^-1^) of BAC		Susceptibility testing medium	Main origin of strains	Reference
		Resistant isolates	Susceptible isolates			
132	12 (9%)	16.0	2.0–8.0	Mueller Hinton agar (MHA) with blood	Poultry (France)	Lemaitre et al., 1998
200	20 (10%)	4.0–7.0	≤2.0	Tryptic soy broth (TSB)	Fish (Norway)	Aase et al., 2000
97	7 (7%)	≥8.0	≤4.0	MHA	Food and others (France)	Mereghetti et al., 2000
19	5 (26%)	≥5.0	≤1.25	TSB	Meat, poultry (USA and Canada)	Romanova et al., 2002
112	17 (15%)	4.0–8.0	2.0–3.0	TSB	Meat (Norway)	Heir et al., 2004
254	108 (42%)	>7.5	≤7.5	MHA	Fish (France)	Soumet et al., 2005
114	9 (8%)	16.0–32.0	4.0	MHA with blood	Retail food (Denmark)	Aarestrup et al., 2007
123	57 (46%)	>10.0	≤10.0	MHA with blood	Poultry (turkey) (USA)	Mullapudi et al., 2008
138	19 (14%)	>10.0	≤10.0	MHA with blood	Different foods (USA)	Ratani et al., 2012
91	15 (16%)	28.0	14.0	MHA with blood	Foods and others (Austria)	Müller et al., 2013
29	3 (10%)	≥10.0	≤2.5	MHA	Pork (Spain)	Ortiz et al., 2014a
71	19 (27%)	≥16.0	<16.0	MHA with blood	Retail foods (China)	Xu et al., 2014
59	13 (22%)	≥12.0	≤10.0	Brain heart infussion (BHI) broth	Retail foods (China)	Jiang et al., 2015
142	25 (18%)	≥10.0	<10.0	MHA with blood	Different foods (Switzerland)	Ebner et al., 2015
20	3 (15%)	≥10.0	<10.0	MHA with blood	Different foods (China)	Zhang et al., 2015
14*^a^*	11 (79%)	≥10.0	≤2.5	MHA	Pork (Spain)	Ortiz et al., 2016

*^a^All from disinfected surfaces.*

Some of these studies have been conducted with a large number of strains, and they report a variable frequency of resistant strains due to the diversity of the selection criteria for the strains studied. For example, the study of Ortiz et al. (2016) reports a very high frequency of resistant isolates because only samples of disinfected surfaces are included (**Table 1**). In some cases, selection for *L. monocytogenes* isolates resistant to QACs has been associated with the repeated use of this class of disinfectants (Heir et al., 2004; Ortiz et al., 2014a, 2016).

In food processing environments, *L. monocytogenes* is exposed to different disinfectants and sanitizers, and sometimes at subinhibitory cocentrations. This is particularly true for disinfectants that are not fully biodegradable which may persist in sewage for long periods. For example, QACs are biodegradable only under aerobic conditions, resulting in continuously fluctuating concentration gradients (Tezel and Pavlostathis, 2015). As a result, the microorganisms are frequently exposed to subinhibitory concentrations of QACs. Repeated exposure to subinhibitory concentrations of QACs and prolonged environmental persistence of certain strains may facilitate the development of resistance over time (Ortiz et al., 2014a). Subinhibitory concentrations of antimicrobials may cause genetic changes in the bacteria by means of different pathways, including an increase in free radicals inside the cell or oxidative stress (Liu et al., 2016). This can trigger the SOS response which can promote either the expression of genes involved in horizontal gene transfer or mutagenesis through induction of the error-prone DNA polymerases (Shapiro, 2015).

Strains of *L. monocytogenes* with genotypic resistance to QACs may have mutations that lead to a reduction in cell permeability (Mereghetti et al., 2000; To et al., 2002). For instance, resistant strains may have modifications in membrane fatty acids and phospholipids (Fox et al., 2011), which can lead to a more anionic and hydrophobic cell surface. This makes it difficult for QACs to pass through the membrane and enter the cell (To et al., 2002).

In other cases, resistance to QACs in *L. monocytogenes* may be due to the acquisition of QAC-specific eﬄux pumps through recombinant elements and mobile genetic elements. Different genetic markers have been identified that confer *L. monocytogenes* with a low-level resistance to QACs, including the resistance determinant *bcrABC* (Elhanafi et al., 2010) and the *qacH* gene of transposon Tn*6188* (Müller et al., 2013), as well as various *qac* determinants originally identified in staphylococci (Xu et al., 2014). All of these genes encode components of the eﬄux system in the small multidrug resistance (SMR) protein family group (Blair et al., 2015). Plasmids are also associated with *L. monocytogenes* resistance to BAC (Elhanafi et al., 2010). Furthermore, plasmids with genes that confer resistance to BAC can be transferred between different pathogenic and non-pathogenic species of *Listeria* in the presence of QACs. This process also leads to the co-selection of resistance against heavy metals (Katharios-Lanwermeyer et al., 2012). In several screening studies of resistant strains, the *qacH* gene and the determinant *bcrABC* have been found (Müller et al., 2013). In one study, for example, resistance to BAC has been associated with the *qacH* gene in the majority (80%) of the tested strains, and a minority of the strains (12%) have been associated with the determinant *bcrABC* (Ebner et al., 2015). Nevertheless, in other studies, only the *qacH* gene has been detected (Ortiz et al., 2014b, 2016), while in others a clear predominance of the determinant *bcrABC* has been reported (Dutta et al., 2013).

On the other hand, in some studies, the aforementioned determinants are not detected in certain resistant strains (Ortiz et al., 2014b, 2016; Ebner et al., 2015). In these cases, resistance may be due to the overexpression of endogenous eﬄux pumps due to mutations in regulatory elements. This can occur due to exposure to QACs or the stress induced by these compounds (Tezel and Pavlostathis, 2015). These pumps are usually chromosomally encoded and affect a broad spectrum of antimicrobial compounds (Buffet-Bataillon et al., 2012; Ortiz et al., 2016). In certain strains of *L. monocytogenes* that are resistant to BAC, QAC resistance has been associated with the overexpression of eﬄux pumps in the MFS (major facilitator superfamily) group (Blair et al., 2015), such as MdrL (multidrug resistant *Listeria*) and Lde (*Listeria* drug eﬄux) (Romanova et al., 2006; Rakic-Martinez et al., 2011). Recently, the overexpression of a new *L. monocytogenes* eﬄux pump (encoded by *emrE*) has been implicated in the resistance to QACs (Kovacevic et al., 2015).

### The Problem of Defining and Detecting Resistance

For various reasons, data on bacterial resistance to biocides is often difficult to interpret and compare (Buffet-Bataillon et al., 2012). A potential reason for the difficulty in interpreting data on bacterial resistance to biocides is the absence of clear criteria for defining a microorganism as resistant to disinfectants. An ecological concept of resistance to biocides has been proposed based on the “natural” susceptibility of a given species (Morrissey et al., 2014). According to this criterion, an isolate is defined as resistant when it is not inhibited by a concentration that would inhibit most of the strains of that particular species. The resistant isolate is typically phenotypically different from the wild type because it has acquired a resistance mechanism through mutation or horizontal gene transfer (EFSA, 2008; SCENIHR, 2009; Buffet-Bataillon et al., 2012; Morrissey et al., 2014). This commonly happens to *L. monocytogenes* strains that are resistant to BAC (Mereghetti et al., 2000; Elhanafi et al., 2010; Müller et al., 2013).

Another reason for the difficulty in comparing results on bacterial resistance is the lack of standardized tests for examining *in vitro* susceptibility to disinfectants (Buffet-Bataillon et al., 2012). Susceptibility to disinfectants is often determined through the MIC technique (usually employing agar dilution or broth dilution methods) (**Table 1**). However, in some cases, the MICs obtained through different methods can produce different results, which is why standardization is necessary. The influence of the culture media is especially important to consider when detecting low-level resistance (**Table 1**). Therefore, we recommend the use of standardized clinical protocols that are designed to evaluate the inhibitory activity of antibiotics (EUCAST, 2000; CLSI, 2009). Consequently, changes in the MIC of the disinfectant of several “test” strains can be measured and the resistant strains can be detected (Ortiz et al., 2014a). In contrast, current standardized tests for the evaluation and comparison of commercial disinfectants measure the bactericidal activity of the products by using one specific “reference” strain (European Committee for Standardisation, 2010; Buffet-Bataillon et al., 2012); the different tests use planktonic cells (suspension test) or cells attached to stainless steel (carrier test).

Despite the low MIC values, *L. monocytogenes* strains with low-level resistance to QACs have eﬄux pumps that can reduce the intracellular concentration of the biocides to subinhibitory levels, “such that the bacterium can survive longer than may have been predicted from the MIC for that organism” (Piddock, 2006). For quinolone-resistant *Staphylococcus aureus*, for example, the MICs afforded by mutations in the gene encoding the target topoisomerase are too low to allow survival, and it has been proposed that eﬄux gives rise to low intracellular concentrations of the drugs (Piddock, 2006). In addition, microorganisms in the environment may be exposed to enormously variable concentrations of QACs. For example, in domestic wastewater the average concentration of QACs is approximately 0.5 mg L^-1^, and in wastewater treatment plant eﬄuents the concentration decreases to 0.05 mg L^-1^ (Tezel and Pavlostathis, 2015). As a result, low-level resistance to QACs may contribute to the persistence of *L. monocytogenes* in the environment.

## Is Persistence Linked to Resistance?

The hypothesis that the persistence of certain subtypes of *L. monocytogenes* is linked to resistance to disinfectants has been investigated in numerous studies (revised by Ferreira et al., 2014). However, an association between low-level resistance to QACs and persistence of the pathogen in different food processing environments has been demonstrated only in a few cases (Aase et al., 2000; Lundén et al., 2003a; Fox et al., 2011; Ortiz et al., 2014a, 2016).

The susceptibility of 200 strains of *L. monocytogenes* to BAC has been investigated in a fish production plant in Norway. In this study, 10% of the strains are resistant strains, and all of these are previously identified persistent strains (Aase et al., 2000; **Table 1**). The relationship between persistence and resistance to two QACs has also been analyzed in four strains of poultry originating in Finland. In one of the persistent strains, the MIC values are higher (2.5–5.0 mg L^-1^) than the MICs for the rest of the strains (0.63–1.25 mg L^-1^) (Lundén et al., 2003a). Additionally, the MICs of a different QAC, benzethonium chloride, have been analyzed in 11 strains of *L. monocytogenes* from the cheese industry in Ireland. Two of the persistent strains have MIC values of 1.5–4.0 mg L^-1^, compared with a MIC of 0.5 mg L^-1^ for the rest of the strains (Fox et al., 2011). In an Iberian pork processing plant in Spain, 29 different subtypes of *L. monocytogenes* have been identified over 3 years, and the resistance to BAC has been associated with the persistence of three subtypes of the molecular serotype 1/2a (Ortiz et al., 2014a). Subsequently, in a similar plant, five different molecular subtypes have been detected in environmental samples from clean, disinfected surfaces (Ortiz et al., 2016). In these eight persistent subtypes, a low-level resistance to QACs has been confirmed. In both of these studies, the MIC values of BAC are 10–20 mg L^-1^ in the resistant strains and 1.25–2.5 mg L^-1^ in the sensitive strains (**Table 1**). To improve the control of *L. monocytogenes* in food processing environments, additional research is needed to evaluate attributes specific to resistant and persistent strains of this pathogen.

## Advantages of Resistant Strains for Persisting in the Environment

**FIGURE 1:**
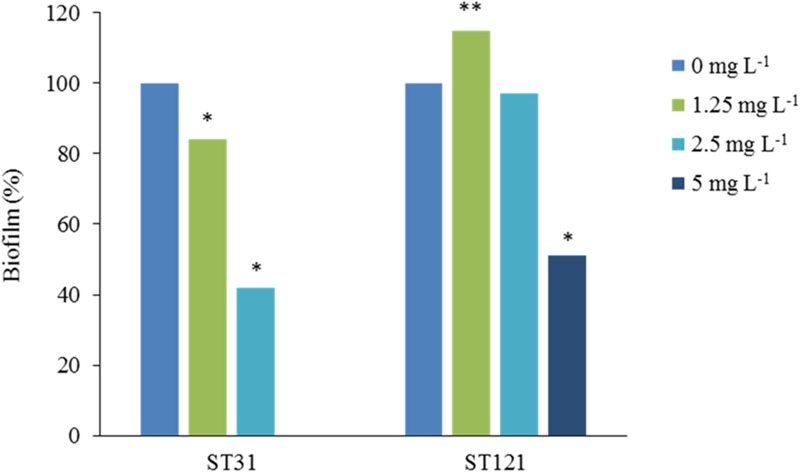
**Effect of BAC on biofilm formation by representative sequence type (ST) 31 and ST121 *Listeria monocytogenes* strains.** The results are presented as the average percentages of three measurements of biofilm formed with the addition of BAC concentrations of 1.25 mg L^-1^, 2.5 mg L^-1^ and 5 mg L^-1^, compared with the control without the addition of BAC (0 mg L^-1^, 100%). Reduced (^∗^) or increased (^∗∗^) percentages of biofilm differed significantly from the controls without BAC (*p* < 0.05, student’s *t*-test), adapted from Ortiz (2015).

The *L. monocytogenes* strains that have acquired mechanisms of resistance to QACs may have specific advantages over sensitive strains with regard to persisting in food processing environments. For example, the influence of residual QACs may vary in sensitive strains and resistant strains, as an optimum concentration (inhibitory) for the former may be suboptimum (subinhibitory) for the latter. This has been confirmed by Ortiz et al. (2014b) in strains of *L. monocytogenes* resistant to BAC. When biofilm formation in the presence of BAC has been studied, only three concentrations of BAC (1.25, 2.5, and 5 mg L^-1^) have been identified that show different effects on the biofilm formation by the group of strains of *L. monocytogenes* included in the study. It was observed that certain resistant strains are able to form biofilm at 5 mg L^-1^, a concentration of BAC that is inhibitory for most strains (Ortiz et al., 2014b). However, biofilm formation in the presence of increased concentrations of BAC may differ between resistant strains with similar MICs but different genetic determinants of BAC resistance. For example, biofilm production in the presence of BAC differs between persistent *L. monocytogenes* strains belonging to sequence type ST121 that have transposon Tn*6188* with the gene *qacH*, and *prfA* mutants belonging to sequence type ST31 that are also persistent and resistant (Ortiz et al., 2016). **Figure 1** shows the percentage of the biofilm formed in the presence of BAC relative to the biofilm formed without BAC by two representative ST31 and ST121 resistant strains. Thus, ST121 strains are able to form biofilm in the presence of BAC concentrations (5 mg L^-1^) higher than the MIC of sensitive strains (1.25–2.5 mg L^-1^) (Ortiz et al., 2016; **Figure 1**). These results may help explain the persistence of the resistant ST121 strains (Ortiz et al., 2014b, 2016; Schmitz-Esser et al., 2015).

Additionally, studies have shown that *L. monocytogenes* strains that are resistant to QACs can form biofilms faster than sensitive strains, which increases the likelihood of their survival (Nakamura et al., 2013). The expression of certain genes associated with the stress response or its regulation may also lead to an increased formation of biofilms by resistant strains (van der Veen and Abee, 2010). Therefore, the exposure of *L. monocytogenes* to subinhibitory concentrations of QACs and the consequent selection of resistant microorganisms may increase the ability of these bacteria to form biofilms and survive future treatment with high concentrations of the same compounds (Tezel and Pavlostathis, 2015).

## Concluding Remarks

In recent years, the evaluation of the relationship between resistance to disinfectants and persistence, have allowed researchers to identify *L. monocytogenes* persistent subtypes that are resistant to disinfectants. The low-level resistance to QACs that has been detected in these subtypes is not a phenotypic adaptation, but rather, it is a stable genotypic resistance that is relatively frequent in certain environments and has important implications. For example, the formation of biofilms by resistant strains in the presence of biocide concentrations that are inhibitory for the sensitive strains may undoubtedly have an effect on their environmental survival. Therefore, a better understanding of the ecological and genetic characteristics of the strains resistant to QACs is needed, as well as standardization and consensus of the techniques used to detect the resistant strains.

## Author Contributions

All authors listed, have made substantial, direct and intellectual contribution to the work, and approved it for publication.

## Conflict of Interest Statement

The authors declare that the research was conducted in the absence of any commercial or financial relationships that could be construed as a potential conflict of interest.

## Abbreviations

BAC: benzalkonium chloride
MIC: minimum inhibitory concentration
QACs: quaternary ammonium compounds
